# Evolution at Spike protein position 519 in SARS-CoV-2 facilitated adaptation to humans

**DOI:** 10.1038/s44298-024-00036-2

**Published:** 2024-07-09

**Authors:** C. Cereghino, K. Michalak, S. DiGiuseppe, J. Guerra, D. Yu, A. Faraji, A. K. Sharp, A. M. Brown, L. Kang, J. Weger-Lucarelli, P. Michalak

**Affiliations:** 1 Department of Biomedical Sciences and Pathobiology, Virginia Tech, Blacksburg, VA USA; 2 Center for Emerging, Zoonotic and Arthropod-borne Pathogens, Virginia Tech, Blacksburg, VA USA; 3 Department of Biomedical Research, Edward Via College of Osteopathic Medicine, Monroe, LA USA; 4 Department of Biochemistry, Virginia Tech, Blacksburg, VA USA; 5 Research and Informatics, University Libraries, Virginia Tech, Blacksburg, VA USA; 6 College of Pharmacy, University of Louisiana Monroe, Monroe, LA USA; 7 Center for One Health Research, VA-MD College of Veterinary Medicine, Blacksburg, VA USA; 8 Institute of Evolution, University of Haifa, Haifa, Israel

**Keywords:** SARS-CoV-2, Viral evolution

## Abstract

As the COVID-19 pandemic enters its fourth year, the pursuit of identifying a progenitor virus to SARS-CoV-2 and understanding the mechanism of its emergence persists, albeit against the backdrop of intensified efforts to monitor the ongoing evolution of the virus and the influx of new mutations. Surprisingly, few residues hypothesized to be essential for SARS-CoV-2 emergence and adaptation to humans have been validated experimentally, despite the importance that these mutations could contribute to the development of effective antivirals. To remedy this, we searched for genomic regions in the SARS-CoV-2 genome that show evidence of past selection around residues unique to SARS-CoV-2 compared with closely related coronaviruses. In doing so, we identified a residue at position 519 in Spike within the receptor binding domain that holds a static histidine in human-derived SARS-CoV-2 sequences but an asparagine in SARS-related coronaviruses from bats and pangolins. In experimental validation, the SARS-CoV-2 Spike protein mutant carrying the putatively ancestral H519N substitution showed reduced replication in human lung cells, suggesting that the histidine residue contributes to viral fitness in the human host. Structural analyses revealed a potential role of Spike residue 519 in mediating conformational transitions necessary for Spike prior to binding with ACE2. Pseudotyped viruses bearing the putatively ancestral N519 also demonstrated significantly reduced infectivity in cells expressing the human ACE2 receptor compared to H519. ELISA data corroborated that H519 enhances Spike binding affinity to the human ACE2 receptor compared to the putatively ancestral N519. Collectively, these findings suggest that the evolutionary transition at position 519 of the Spike protein played a critical role in SARS-CoV-2 emergence and adaptation to the human host. Additionally, this residue presents as a potential drug target for designing small molecule inhibitors tailored to this site.

## Introduction

Severe acute respiratory syndrome coronavirus 2 (SARS-CoV-2) is the causative agent of coronavirus disease 2019 (COVID-19), which has claimed just under seven million lives and caused 770 million cases of the disease since its emergence in 2019^1^. SARS-CoV-2 is a positive sense, single-stranded RNA virus of the family *Coronaviridae*, genus *Betacoronavirus*, and subgenus *Sarbecovirus*^2^. The first human cases were detected in the city of Wuhan in the Hubei province of China^3,4^ and were associated with the Huanan wholesale seafood market^5,6^, where the virus may have spilled over from a live animal source^7,8^.

While not definitive, there is evidence of a bat origin of the progenitor to SARS-CoV-2^9^. Sarbecoviruses isolated from bats have high nucleotide sequence similarity to SARS-CoV-2 on a genome level and within the receptor-binding domain (RBD) of the Spike protein^4,10^. BANAL-20-52, isolated from a bat in Laos has the highest nucleotide similarity with SARS-CoV-2 to date at 96.8%^10^. Hypotheses posited the progenitor to SARS-CoV-2 evolved in bats and was pre-adapted to humans since positive selection was detected on deep branches of the nCoV clade and not terminal branches leading to SARS-CoV-2 on a phylogenetic tree^9,10^. However, the precise molecular mechanisms of adaptation of the progenitor to SARS-CoV-2 to humans have not been well characterized despite how these data would aid in the development of therapeutics and surveillance for pandemic prevention. Our previous study determined evolution towards an alanine at position 372 in the SARS-CoV-2 Spike protein was important for human adaptation by first identifying selective sweeps in the SARS-CoV-2 genome^11^. Selective sweeps occur when a favorable mutation is quickly fixed in the population^12^. Neighboring “hitch-hiker” alleles also increase in frequency as a result of the driver mutation of the selective sweep on the recombinant genomic region^13,14^. Thus, parsing out truly adaptive mutations requires experimental validation and is critical to determining mechanisms of emergence.

Many studies investigating the emergence of SARS-CoV-2 have strictly involved computational analyses or the generation of pseudotyped viruses expressing Spike. These methods are useful but do not validate predictions or provide the most accurate quantitation of fitness of SARS-CoV-2 compared to examining phenotypes of replication-competent virus. Here, we gain new insights into the evolutionary pressures and molecular mechanisms behind the emergence of SARS-CoV-2 in humans by generating both pseudotyped and replication-competent virus with a putative ancestral residue mapping to a selective sweep region, as based on the analysis of approximately two million SARS-CoV-2 genomes. The mutation (Spike H519N) significantly decreased SARS-CoV-2 replication in human lung epithelial cells and reduced infectivity in pseudotyped virus assays and binding to human ACE2 in a biochemical assay, consistent with structural predictions. This study provides evidence that the evolution at Spike site 519 was important for the progenitor of SARS-CoV-2’s adaptation to the human host. The Spike site also remains highly conserved among SARS-CoV-2 sequences and offers a suitable candidate for targeting with small molecule inhibitors.

## Results

### SARS-CoV-2 sequences hold evidence of a past, strong selective event in the receptor binding domain of Spike

**Fig. 1:**
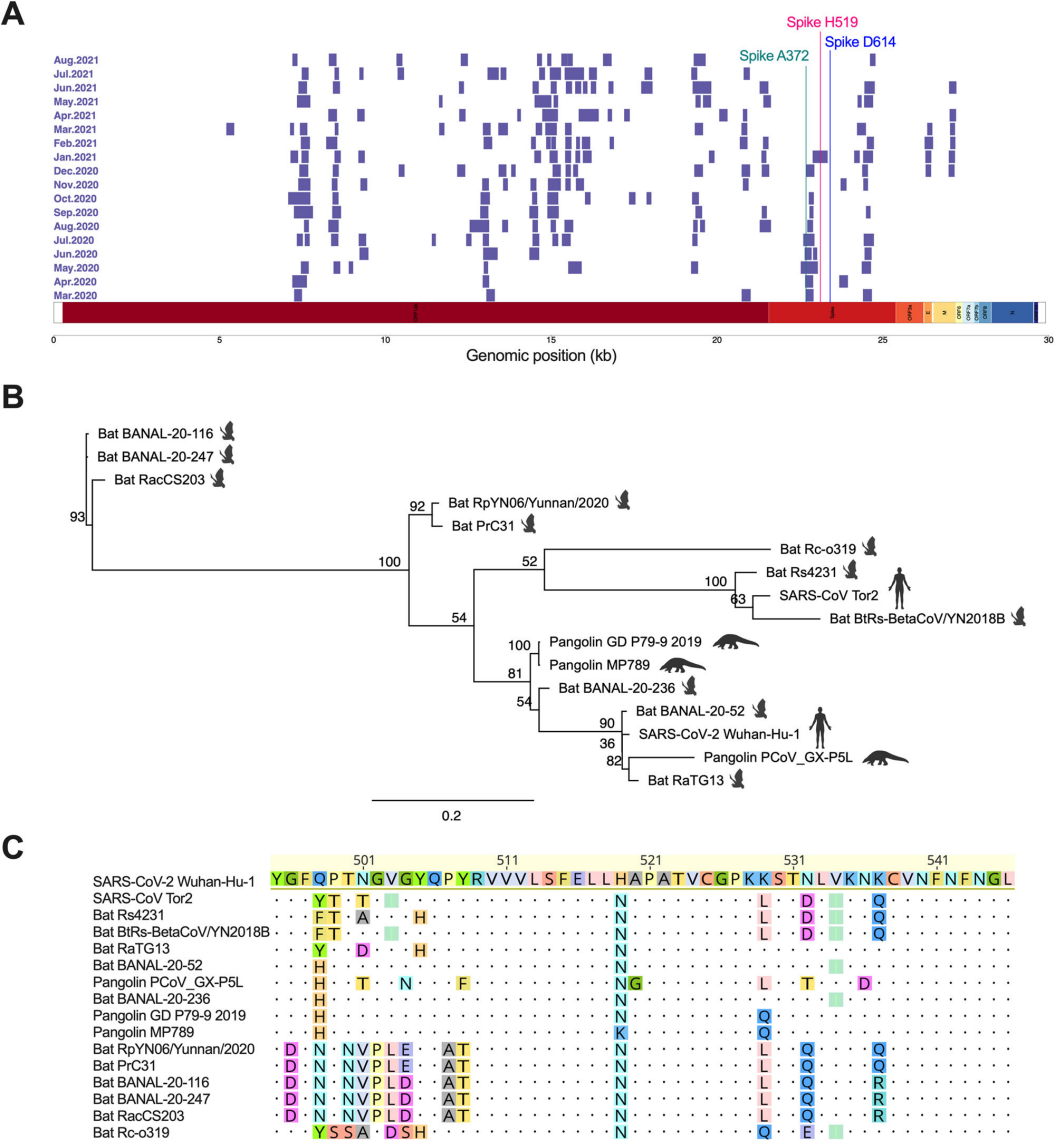
Analysis of millions of SARS-CoV-2 sequences identified selective sweep regions within Spike. **A** Selective sweep regions in 2 million SARS-CoV-2 genomes were identified per month up to August 2021 to identify signatures of recent positive selection during the initial stages of the pandemic. Sweep regions are denoted in purple. Results are summarized for the 2 million sequences. Highlighted sites correspond to the site of interest and positive controls Spike T372A and Spike D614G. **B** Phylogenetic tree of SARS-CoV-2 and SARS-related coronaviruses constructed from the Spike amino acid sequence using PhyML LG substitution model and 1000 bootstraps. The species from which the virus was isolated is depicted by an image of the animal to the right of the virus name. **C** Amino acid alignment of sweep region in SARS-CoV-2 RBD with corresponding region in closely related sarbecoviruses from bats and pangolins and SARS-CoV. The alignment was performed using MAFFT.

To investigate which regions in SARS-CoV-2 have contributed to its efficient infection and circulation in humans during the initial stages of the pandemic, we analyzed sequences for signs of strong positive selection events known as selective sweeps. We acquired 1,914,191 human-isolated SARS-CoV-2 sequences from GISAID, covering the period up to August 2021 to constitute an early period of the pandemic. We then used a computational pipeline combining OmegaPlus^15^ and RAiSD^16^ to identify regions with a high probability of selective sweep events. To discern the selective sweep regions instrumental in driving mutations during the early stages of human infection, we categorized the sequences by their sample collection dates, arranging them into monthly cohorts (Fig. 1A). Sweep regions containing Spike D614G and Spike T372A, two human-adaptive mutations^11,17^, were examined to ensure the reliability and accuracy of our methods. Our analysis identified an average of 11 sweep regions per month, with the numbers ranging from 4 to 16 (Supplementary File 1). While many sweep regions were identified, our attention was primarily drawn to the sweep regions in the receptor-binding domain (RBD) of the Spike protein (nucleotide position 22,517 to 23,185; amino acid positions 319-541), as mutations in the RBD are known modulators of host tropism and receptor binding^18^. The selective sweep regions included the A372 residue in the RBD, a critical factor we previously identified for the emergence of SARS-CoV-2 and its sustained transmission among humans^11^. These regions were evident in the early months of the COVID-19 outbreak (Fig. 1A).

Since selective sweep regions indicate a past selective event driven by various adaptive mutations and their associated hitchhiker mutations, we sought to determine the site within the Spike selective sweep region where pivotal mutations occurred upon early infection of humans^19^. While the progenitor virus to SARS-CoV-2 is unknown, we inferred the progenitor Spike sequence could assume several amino acid identities found at the corresponding site in Spike from closely related sarbecoviruses infecting bats and pangolins. To identify the closest coronaviruses related to SARS-CoV-2, we constructed a phylogenetic tree based on the amino acid sequence of Spike with PhyML (Fig. 1B)^20,21^. Using these phylogenetic relationships, we aligned the amino acid sequence of the selective sweep region in Spike from the Wuhan-Hu-1, lineage B strain of SARS-CoV-2 with the homologous region in closely related sarbecoviruses to reveal sites with differential amino acid identities (Fig. 1C; see also Supplementary Fig. 1 for the full amino acid alignment of the sweep region in Spike). Similar to amino acid position 372 in Spike, position 519 holds a non-synonymous mutation that differentiates SARS-CoV-2 from the aligned bat- and pangolin-derived *Sarbecovirus* sequences^11^. This position was identified in the selective sweep regions during one of the early months of the outbreak (January 2021; Fig. 1A). SARS-CoV-2 has a histidine at 519, while the bat and pangolin-derived sequences bear an asparagine or lysine. We hypothesized the progenitor to SARS-CoV-2, potentially a virus infecting bats or pangolins, acquired a histidine at some point in the evolutionary timeline, thereby gaining an adaptive advantage in humans.

Since sites with low amino acid diversity may implicate a crucial role of the predominant amino acid in viral fitness, we sought to investigate the amino acid diversity at position 519 in Spike in SARS-CoV-2. Using data from 3848 genomes sampled between December 2019 and October 2023 from the nCoV GISAID dataset displayed on Nextstrain, we determined Spike position 519 in SARS-CoV-2 has a normalized Shannon entropy of 0, suggesting little to no flexibility is allowable at this position in humans (Supplementary Fig. 2)^22,23^. With this result, we hypothesized the predominant histidine at Spike 519 in SARS-CoV-2 may hold an important function for the virus in humans, while the ancestral residue, asparagine, would result in deleterious effects on fitness in human cells.

### SARS-CoV-2 Spike mutant bearing a residue of bat and pangolin *Sarbecovirus* origin has reduced replicative fitness and infectivity in human cells

**Fig. 2:**
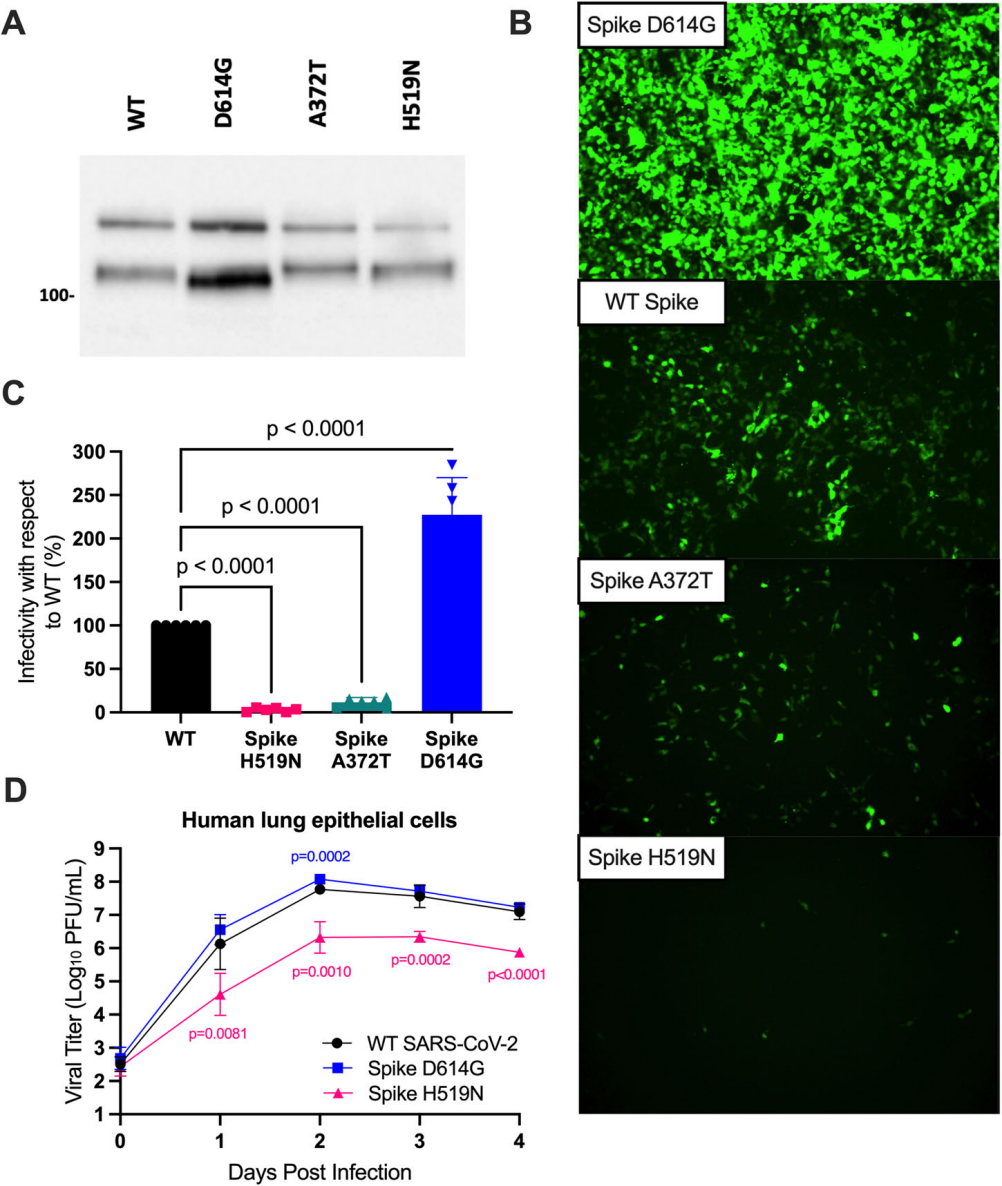
Decreased replicative fitness and infectivity of SARS-CoV-2 Spike H519N in human lung and kidney cells. **A** Western blot analysis of Spike protein from prepared pseudovirus stocks. An antibody was used to detect the S1 subunit of Spike. The higher band at 180 kilodaltons (kDa) corresponds to full Spike while the band near 100 kDa corresponds to furin-cleaved Spike. Units are represented in kDa. **B** Representative images of Zsgreen expression in Spike pseudotyped virus infected HEK-293T-hACE2 cells observed by live-cell fluorescence microscopy. Cells infected with pseudoviruses expressing ZsGreen and luciferase pseudotyped with full Wuhan-Hu-1 Spike or mutants Spike D614G, A372T, or H519N. Images were taken 48 h post-infection and are representative of one of two technical replicates from three independent experiments. **C** Infectivity was quantified using a luciferase assay 48 h post-infection. Experiments were performed in duplicate for three biological experiments. Statistical analyses were performed using a one-way ANOVA with Dunnett’s correction for multiple comparisons. **D** Growth curve of Spike H519N in human lung epithelial cells. Calu-3 cells were infected at a MOI of 0.1 with the WT Wuhan-Hu-1 strain of SARS-CoV-2 and mutants bearing either Spike H519N or D614G. Supernatant was collected each day post-infection and titered on Vero E6-TMPRSS2-T2A-hACE2 cells by plaque assay. Infections were performed in triplicate in two independent biological experiments. A two-way ANOVA with Dunnett’s correction for multiple comparisons was performed on these data. Bars represent standard deviation.

Towards identifying the driving mutations of the putative selective event, we reasoned evolution from the asparagine to histidine at position 519 may have driven early adaptation of the progenitor virus to humans. Since position 519 is in the RBD of Spike, we first used a previously described pseudovirus system^24^ to determine whether Spike H519N had any functional significance during infection of cells expressing human ACE2 (hACE2), the receptor for SARS-CoV-2^25^. First, we verified hACE2 protein levels by western blot expressed in human embryonic kidney cells (Supplementary Fig. 3). We generated lentiviruses pseudotyped with full wild-type (WT) Spike, Spike H519N, Spike D614G, and Spike A372T, a RBD mutant bearing an ancestral threonine which we previously showed attenuates the virus in human lung epithelial cells^11^. Spike D614G is used as a known human-adaptive variant that emerged early in the pandemic and is now fixed in all SARS-CoV-2 variants^17^. The pseudoviruses express both luciferase and a green fluorescence protein (GFP), ZsGreen^24^, allowing for sensitive detection of infection efficiency. We detected Spike protein levels from prepared virus stocks and observed a greater incorporation of G614 into the pseudoviruses as previously observed (Fig. 2A)^26^. Ectopic expression of Spike was similar between WT Spike and Spike H519N. Next, we used pseudovirus particles to infect human embryonic kidney cells expressing hACE2 to determine whether infectivity through hACE2 is altered by Spike H519N. Spike D614G enhanced the infectivity of the pseudotyped viruses compared to WT Spike, in agreement with data extensively reported in literature (Fig. 2B)^26^. Spike A372T significantly reduced the infectivity of SARS-CoV-2, consistent with our previous study (Fig. 2B)^11^. Importantly, Spike H519N significantly reduced the infectivity of SARS-CoV-2 compared to Spike D614G and WT Spike (Fig. 2B). When infected cells were quantified based on luciferase expression, we also observed significant decreases in infectivity for Spike H519N compared to WT Spike (Fig. 2C; *p* < 0.0001).

Towards determining whether Spike N519 reduces the replicative fitness of SARS-CoV-2 in human cells, we generated a replication-competent, SARS-CoV-2 mutant bearing an asparagine at position 519 in Spike, the amino acid present in closely related sarbecoviruses. In human lung epithelial cells, the putatively ancestral SARS-CoV-2 Spike H519N mutant replicated to significantly lower titers than the WT virus and Spike D614G, demonstrating a nearly 2-log difference in viral titers (Fig. 2D; *p* < 0.01 at all timepoints). These data suggest that reverting the histidine at Spike 519 to the ancestral asparagine significantly reduces infection and replication in human lung epithelial cells.

### Spike H519N potentially impacts conformational transitions and reduces binding affinity to human ACE2

**Fig. 3:**
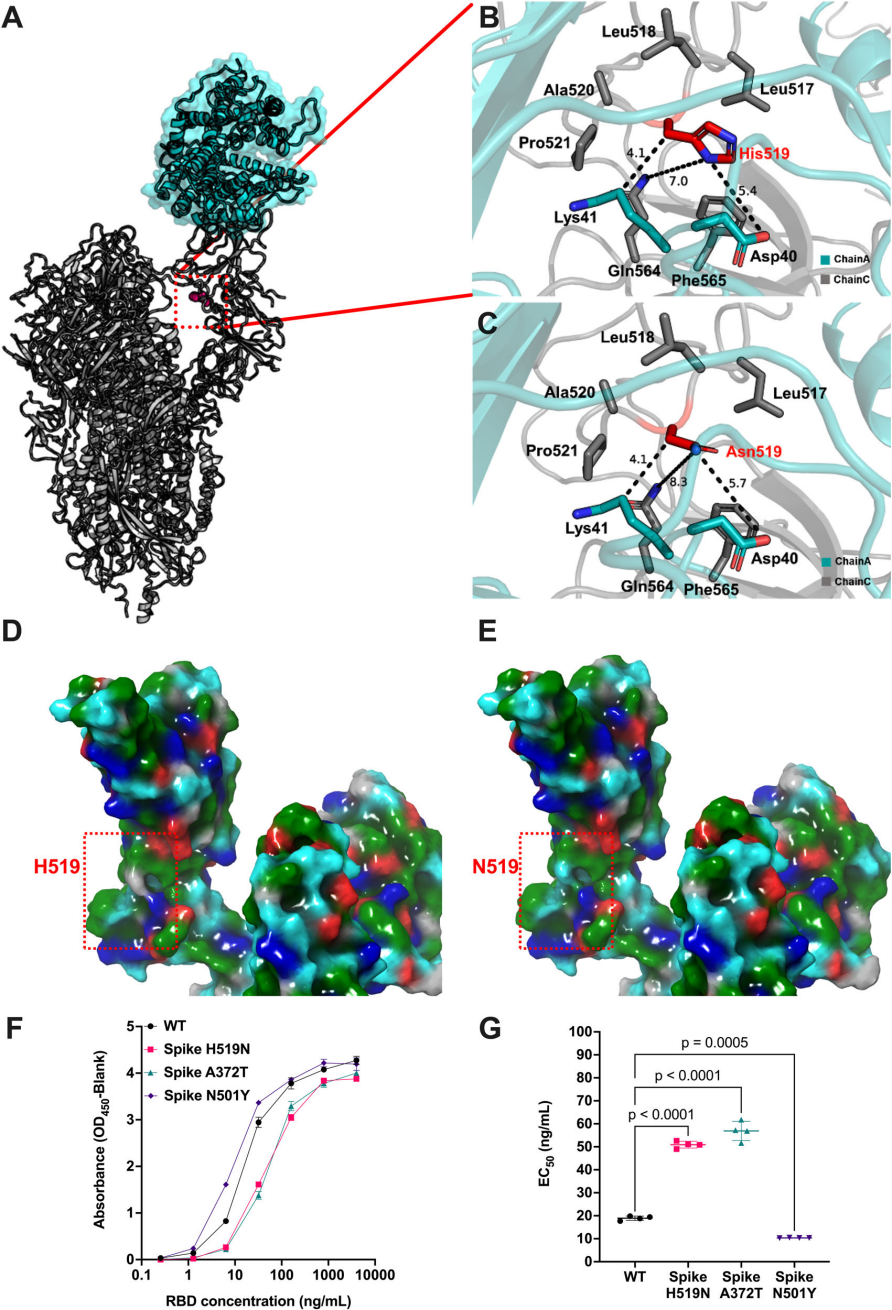
Structural and biochemical analyses of Spike H519N reveal decreased affinity to human ACE2. **A** Structure of Spike bound to hACE2 (PDB: 7KNB). 519 location is represented in pink spheres**. B** Visualization of H519 and **C** N519 structural location. 519 is highlighted in red, with residues within the same chain in gray and a neighboring chain in teal. **D** Surface mapping of Spike H519 and **E** N519 colored by residue sidechain properties. Colors represent gray for neutral, teal for polar uncharged, blue for positive charge, red for negative charge, and green for hydrophobic. **F** ELISA saturation curves. Human ACE2-mFc was coated at 2 μg/mL, and various concentrations of Spike RBD were detected with an antibody. Absorbance values were measured at OD_450_nm, and the values normalized to the blank are depicted. Curves intersect the mean of four binding events. **G** EC_50_ concentrations from ELISA curves extrapolated with a nonlinear regression with a least squares fit. A one-way ANOVA with Dunnett’s Correction was used to test for statistical significance.

In elucidating the mechanism of attenuation of SARS-CoV-2 bearing the putatively ancestral asparagine at Spike position 519, we analyzed the interactions between Spike chains to identify how H519N is impacting infection (Fig. 3A). Residue 519 is in the RBD but not within the receptor binding motif (RBM) to interact with ACE2 directly. Rather, residue 519 is positioned on a cleft at the interface between Spike chains that constitute the full trimer complex. Indeed, residue 519 is within 4–5 Å of residues of adjacent chains of the Spike trimer, participates in polar interchain interactions, and conformational up/down movement can be impacted at this interface. Here, we sought to utilize this interaction interface to compute interchain interaction energy via MM/GBSA calculations analyzing up/down conformation favorability by probing interchain interaction energy in Spike. Results demonstrate that the H519 up conformation has similar interchain interaction energy to the N519 up conformation when unprotonated (−284.1 kcal/mol and −264.1 kcal/mol, respectively). The down conformation exhibits larger differences between the H519 and N519 structures, with H519 interchain interaction energy of −259.2 kcal/mol and N519 of −365.9 kcal/mol. Interestingly, when analyzing protonated H519, we observe a predicted interchain interaction energy for the up conformation H519 of −359.4 kcal/mol and −280.9 kcal/mol for the down confirmation of H519. This suggests that in a lower physiological pH, the H519 Spike samples the up conformation more favorably compared to the N519, which alternatively energetically favors the down conformation based on interchain interactions. Structural analysis of the neighboring residues reveals that 519 is surrounded by a pocket of polar and charged residues from a neighboring Spike chain (Fig. 3B, C). Surface mapping of residue properties highlights that H519 exhibits a neutral surface area (Fig. 3D) while N519 results in a more polar surface area (Fig. 3E). These results suggest that H519 may result in a lower energy barrier to overcome when transitioning between down/up positioning to bind ACE2, potentially allowing for increased infection efficiency.

With the insight that H519N appears to impact conformational transitions in protonated SARS-CoV-2 Spike and the potential ability to bind ACE2 by sampling the up position more favorably, we further sought to determine whether Spike H519 would experimentally bind with higher affinity to hACE2. Enzyme-linked immunosorbent assay (ELISA) was performed with hACE2 and several concentrations of RBD from either WT Spike, Spike N501Y, Spike A372T, or Spike H519N. Spike N501Y was used as a positive control with known increased binding to hACE2^27^. Consistent with reduced binding efficiency, the absorbance curve for Spike H519N and Spike A372T RBD are shifted right from WT RBD (Fig. 3F). We observed significantly lower EC_50_ values for Spike N501Y RBD and significantly higher EC_50_ values for Spike H519N and Spike A372T compared to WT RBD (Fig. 3G). This result suggests more molecules of Spike H519N RBD would be required to saturate hACE2 binding sites; therefore, this mutation may reduce the affinity to hACE2 leading to reductions in replication.

## Discussion

During zoonotic spillover events, selective pressures exist for pathogens to adapt to the cellular components and immune responses of new host species in the pursuit of optimal fitness. RNA viruses like SARS-CoV-2 are skilled at navigating the fitness landscape in new hosts, taking advantage of both recombination and the error-prone RNA-dependent RNA polymerase to source new genotypes that can lead to fitness advantages^28^. Our study provides evidence of historical selection acting on SARS-CoV-2 via selective sweeps and assesses the contribution of an amino acid site within a selective sweep region in the RBD of Spike that likely facilitated adaptation to humans. We provide evidence that the putatively ancestral asparagine at Spike 519 in closely related sarbecoviruses reduces the fitness of SARS-CoV-2 in cells expressing hACE2 due to reduced affinity of Spike RBD N519 to hACE2 and higher binding free energy in the up conformation.

Several studies have gathered strong evidence for a natural origin of SARS-CoV-2, but there is disagreement on how SARS-CoV-2 might have evolved to sustain productive infection of and transmission between humans. Some studies have suggested the progenitor to SARS-CoV-2 evolved in bats and required little to no adaptation to humans before spillover and emergence^10^. While we do not refute evolution of the progenitor to SARS-CoV-2 occurred in bats, our study provides evidence of a mutation that enhanced human infection but does not predict in which host this mutation arose. It is possible that genetic drift led to mutations in the reservoir host which enabled human adaptation prior to human exposure^9^. Furthermore, our study indicates the transition at Spike 519 from N to H in SARS-CoV-2 evolution led to a gain of fitness in humans, but a fitness tradeoff in putative ancestral host species is not necessarily implicated nor required for adaptation of Spike H519 to humans to remain true. For example, transitions in RBD residues of SARS-CoV isolated from a palm civet to the homologous residue in human-isolated SARS-CoV sequences resulted in a gain of function with infection via hACE2 but no corresponding fitness loss with infection via palm civet ACE2^18^. Therefore, viral adaptation to one species does not require a fitness loss in another host. This study is the second of ours that supports the theory that the progenitor to the Wuhan-Hu-1 strain of SARS-CoV-2 was subject to a strong selective pressure resulting in adaptation to humans at a site in the RBD of Spike^11^.

Zoonotic spillover events are often associated with the selection of mutations in viral receptor binding proteins that afford the use of orthologous receptors in the human host^29^. For coronaviruses in particular, mutations in the Spike protein, which binds the host ACE2 receptor, are sufficient to expand the host range of the virus even though a variety of other factors are important for tropism^18^. Another zoonotic coronavirus, severe acute respiratory syndrome coronavirus (SARS-CoV), which caused an epidemic from 2002-2003, emerged likely due to a set of mutations both in the receptor binding domain (RBD) and other parts of Spike and underwent adaptive evolution from its reconstructed ancestral sequence^18,30^. Our results are in agreement with other studies that mutations outside of the RBM of the coronavirus Spike protein, including both subunits of Spike, can affect receptor binding and are also associated with host-range expansion^11,31,32^.

Amino acid position 519 in Spike is in the RBD but not within the receptor binding motif (RBM). The putatively ancestral asparagine has more favorable co-association of Spike in the down conformation, which impacts the potential to transition to an up conformation and bind with hACE2. This can be observed with the reduced binding affinity of Spike H519N RBD to hACE2 via ELISA and interchain interaction energy calculations highlighting the H519 up conformation favorability in an acidic environment. While these results provide insight into potential structural mechanisms related to the fitness of H519, more robust molecular dynamics (MD) simulations could reveal more major morphological changes related to 519 fitness in transiting between up/down conformations. Our results are not surprising since mutations outside of the RBM have been shown to increase binding affinity to hACE2 during a deep mutational scan of the Spike RBD^33^, and our previous work indicates Spike 372 influences hACE2 binding^11^. Furthermore, Spike 614, which lies outside of the RBD, affects ACE2 binding^34^. Previous work has indicated the structural region surrounding residue 519 is involved in pH sensing^35^ and structural transition states^36^ which can also ultimately affect infectivity. Our results are biologically relevant given that the human nasal cavity pH is 6.6 which might provide a more optimal environment for the protonated SARS-CoV-2 Spike H519 to associate more with hACE2 than Spike N519 in this acidic environment^37^. Since transmission of coronaviruses between bats is suspected to primarily occur via the fecal-oral route^38,39^, the microenvironment of the bat gastrointestinal tract rather than the bat nasal cavity likely has more influence on coronavirus transmission between bats. More research is needed on this microenvironment and how it may shape bat coronavirus evolution. Taken in an evolutionary context, it is possible that Spike N519H arose in the progenitor virus due to stochastic or deterministic processes and that this mutation increased association with hACE2 when the virus initially encountered humans, leading to enhanced infection and transmissibility.

We used Nextstrain to examine the diversity within Spike and identified a low normalized Shannon entropy of 0 at position 519 in Spike of SARS-CoV-2 sequences from 2019-2023 from GISAID. Since we have shown the histidine at position 519 in Spike is important for replication in human lung cells and that it is highly conserved regardless of lineage and variant of concern, position 519 might constitute a suitable drug target. Indeed, structural inspection of the surface area maps and structural residue region of 519 indicates that it is within a solvent accessible pocket, surrounded by polar and charged residues from neighboring chains. Suitable pockets for small molecule inhibitors may also correlate with regions of low diversity in the genome, however further investigation is needed. Efforts to design small molecule inhibitors of SARS-CoV-2 have resulted in targeting genes such as the main protease of SARS-CoV-2^40^. Recent surveillance studies identified multiple mutations in SARS-CoV-2 that confer resistance to nirmatrelvir^41^. Another protease inhibitor ensitrelvir is being used for emergency use authorization in Japan, but mutations conferring resistance to this inhibitor have been identified as well^41^. This highlights the importance of finding new components of the virus to target, and the RBD at position 519 in Spike might be suitable since other amino acids at this position have not emerged to any appreciable extent.

To conclude, a mutation altering the amino acid identity at position 519 in the Spike protein of SARS-CoV-2 is likely to have partly mediated adaptation of the progenitor virus to the human host. We demonstrated the conserved histidine at position 519 in Spike affords fitness advantages through increased replication in human cells via increased hACE2 binding affinity. Position 519 in Spike holds promise as a site to which small molecular inhibitors could be designed amidst the concern of the development of drug resistance.

## Methods

### Selective sweep analysis of SARS-CoV-2 genomes

A total of 1,914,191 human-derived, complete SARS-CoV-2 genomes up to Aug 2021 were downloaded from the GISAID EpiCov database (https://www.gisaid.org/). Sequences that had low coverage or displayed over 5% nucleotide ambiguities (designated as ‘N’) were excluded from the analysis to ensure analytical rigor. For further analysis, sequences were categorized monthly based on their sample collection dates. OmegaPlus and RAiSD were used with these data for the determination of selective sweep regions. OmegaPlus^15^ is an implementation of the ω statistic^42^ for whole-genome data, which takes into account linkage disequilibrium, whereas RAiSD takes into account three signatures of selective sweeps^16^. Selective sweep identification was conducted within these monthly cohorts using previously established data curation and methodologies^11^.

### Construction of phylogenetic tree

The following coronavirus sequences were downloaded from GenBank: Bat coronavirus isolate BANAL-20-52/Laos/2020 MZ937000.1, Bat coronavirus isolate BANAL-20-103/Laos/2020, complete genome MZ937001.1, Bat coronavirus isolate BANAL-20-116/Laos/2020, complete genome MZ937002.1, Bat coronavirus isolate BANAL-20-236/Laos/2020, complete genome MZ937003.2, Bat coronavirus isolate BANAL-20-247/Laos/2020, complete genome MZ937004.1, Bat coronavirus RacCS203, complete genome MW251308.1, Bat coronavirus RaTG13, complete genome MN996532, Bat coronavirus strain BetaCoV/Rm/Yunnan/YN02/2019 spike protein (S) gene, partial cds MW201982.1, Bat sarbecovirus sp. isolate PrC31, complete genome MW703458.1, Bat SARS-like coronavirus isolate Rs4231 KY417146.1, Bat Severe acute respiratory syndrome-related coronavirus Rc-o319 RNA, complete genome LC556375.1, Betacoronavirus sp. RpYN06 strain bat/Yunnan/RpYN06/2020, complete genome MZ081381.1, Coronavirus BtRs-BetaCoV/YN2018B MK211376.1, MAG Pangolin coronavirus isolate GD P79-9 2019, complete genome OQ297708.1, Pangolin coronavirus isolate MP789, complete genome MT121216.1, Pangolin coronavirus isolate PCoV_GX-P4L, complete genome MT040333.1, Pangolin coronavirus isolate PCoV_GX-P5L MT040335.1, SARS coronavirus Tor2 AY274119.3, Severe acute respiratory syndrome coronavirus 2 isolate Wuhan-Hu-1 NC_045512.2. Geneious (version 2023.1 created by Biomatters. Available from https://www.geneious.com) was used to construct a phylogenetic tree based on the amino acid sequence of Spike using PhyML 3.3.20180214 with a LG substitution model and 1000 bootstraps^20^. An amino acid alignment of the selective sweep region in Spike identified in January 2021 was performed using Geneious with the same viruses as above to identify non-synonymous mutations compared to the Wuhan-Hu-1 strain of SARS-CoV-2.

### Diversity analysis in Spike

Nextstrain was accessed on November 1, 2023 displaying 3848 nCov genomes sampled between December 2019 and October 2023 from the GISAID nCoV dataset. Amino acid diversity data was obtained by downloading the full Spike diversity dataset from the site. Nextstrain calculates normalized Shannon entropy by summing the products of the amino acid frequency at a given position and the natural log of the frequency for each amino acid observed. The frequency is normalized by the number of tips in the phylogenetic tree on Nextstrain^22^.

### Cell lines and plasmids

Human embryonic kidney cells expressing human ACE2 (HEK-293T-hACE2; NR-52511) and African green monkey kidney epithelial cells expressing TMPRSS2 and human ACE2 (Vero E6-TMPRSS2-T2A-hACE2; NR-54970) were obtained from BEI Resources. Human lung epithelial cells (Calu-3 HTB-55) and human embryonic kidney cells (HEK-293T) were acquired from ATCC. Cells were grown in a humidified atmosphere with carbon dioxide supplied at 5% at 37 °C. HEK-293T and HEK-293T-hACE2 cells were grown in Dulbecco’s modified Eagle medium (DMEM; Corning™ 10013CV) supplemented with 10% Fetal Bovine Serum, 100 U/mL penicillin, 100 U/mL streptomycin, 2 mM L-glutamine. Vero E6-TMPRSS2-T2A-hACE2 and Calu-3 cells were grown in Dulbecco’s modified Eagle medium supplemented with gentamicin sulfate (0.1%), non-essential amino acids (1X), HEPES (25 mM), and either 5% (Vero E6-TMPRSS2-T2A-hACE2) or 20% (Calu-3) fetal bovine serum. Vero E6-TMPRSS2-T2A-hACE2 also required the addition of 0.01 mg/mL puromycin. SARS-Related Coronavirus 2, Wuhan-Hu-1 Spike-Pseudotyped Lentiviral Kit V2 was obtained from BEI Resources (NR-53816).

### Generation of Spike pseudotyped virus mutants

Spike mutations A372T and H519N were introduced to the human codon-optimized Spike gene on vector pHDM SARS-Related Coronavirus 2 Wuhan-Hu-1 Spike Glycoprotein (BEI; NR-53742). We used the BioLabs Q5 Site-Directed Mutagenesis Kit (NEB #E0554) as described from the manufacturer’s protocol with mutagenesis primers Forward (CGAATTGCTCAACGCTCCAGC) and Reverse (AAACTCAAGACCACAACTCTG) for mutant H519N, and Forward (GTATAATAGTACAAGCTTTAGCACATTC) and Reverse (AATACTGAGTAGTCCGCC) for mutant A372T. Mutations were confirmed by Sanger sequencing. Vector HDM SARS2-Spike-del121 D614G was purchased from Addgene (cat #158762). Pseudotyped lentiviral particles were generated by transfecting HEK-293T cells using PolyJet In Vitro DNA Transfection reagent (SignaGen cat #SL100688) following the manufacturer’s recommendations for the six-well plate, with the adjusted amount of 3 µg for each of the following BEI plasmids: pRC-CMV-Rev1b (NR-52519), HDM-tat1b (NR-52518), HDM-Hgpm2 (NR-52518), Luciferase-IRES-ZsGreen (NR52516), and 3 µg of the pHDM vector encoding the Wuhan-Hu-1 Spike Glycoprotein (WT), or D614G, A372T, or H519N spike mutations, respectively. We monitored the transfection efficiency by detecting ZsGreen signal via live cell fluorescence microscopy using a Leica DMI4000B inverted microscope. Supernatants with lentiviral particles were harvested 48- and 72-h post-transfection. Pseudoviruses were precipitated overnight at 4 °C with 40% PEG-8000/1.2M NaCl pH 7.2 at a 1 to 3 ratio, centrifuged at 1200 rpm for 1 h at 4 °C, and obtained pellet resuspended in 1/10 of the original collection volume.

### Western blot analysis of pseudotyped virus Spike incorporation

We verified the presence of SARS-CoV-2 Spike protein on WT and mutated pseudotyped lentiviral particles by Western blot analysis. Viral protein lysates were denatured at 95 °C, resolved on a 10% SDS-PAGE gel, and transferred to Biorad Immun-Blot PVDF membrane (cat #1620177). After 1 h blocking in 5% milk in TBS-T buffer (50 mM Tris [pH 7.5], 150 mM NaCl containing 0.1% Tween 20), the membrane was incubated overnight with Invitrogen Anti-SARS-CoV2-Spike protein S1 primary antibody (cat #PA5-114528). After three washes with TBS-T buffer, blot was probed for 1 h with Peroxidase-conjugated AffiniPure Goat Anti-Rabbit IgG (H + L) secondary antibody (Jackson ImmunoResearch Laboratories cat #115-035-003) in 5% milk/TBS-T buffer. The membrane was washed three times with TBS-T, and the signal was detected with Biorad Clarity Western ECL substrate cat (#170-5061) and imaged using Biorad ChemiDoc MP Imaging System.

### Quantification of SARS-CoV-2 plasmid pseudogenome via real-time PCR

For the quantification of pseudovirus particles, we extracted the DNA from lentiviral stocks using Qiagen DNeasy Blood & Tissue Kit (cat #69504) according to the manufacturer’s protocol. We quantified a ZsGreen amplicon by qPCR via Applied Biosystems PowerSYBR Green PCR Master Mix (cat #4376659) using Forward (GACCATGAAGTACCGCATG) and Reverse (CCGCCCTCCACCACGCAC) ZsGreen-specific primers. The DNA Standard curve of 10, 1, 0.1, and 0.01 ng/µL serial dilutions of pHAGE-CMV-Luc2-ZsGreen plasmid served as our reference. The relative amounts of pseudoviral genomes of all mutants were adjusted to WT lentivirus before infections.

### Luciferase assay

The luciferase activity of HEK-293T-hACE2 cells infected with pseudovirus was measured using the Promega Luciferase Assay System according to the manufacturer’s protocol (cat #E1500). Cells were washed 2 times with PBS and lysed in 1X Lysis Buffer per well in a 12-well plate. 100 µL of the 1X Luciferase Assay per 20 µL of lysate was used for detection of luciferase activity in each well of a 96-well black microplate. The efficiency of infection was determined by measuring the intensity of luminescence calculated as relative luciferase units using the Agilent BioTek Synergy HTX Multi-Mode Microplate Reader.

### Generation of SARS-CoV-2 mutants

Live SARS-CoV-2 mutants were generated from a previously constructed full-length infectious clone of the Wuhan-Hu-1 strain of SARS-CoV-2 (GenBank accession no. NC_045512.2). Mutagenic primers were used to introduce mutations into the backbone by PCR with Invitrogen Platinum SuperFi II PCR master mix (12368010) or Quantabio repliQa HiFi ToughMix (95200-025). Fragments were purified from agarose gels using the Machery-Nagel nucleospin gel and pcr clean-up kit (740609.250). Purified fragments were mixed at an equimolar ratio and assembled and amplified by replication cycle reaction using the OriCiro Genomics Cell-Free Cloning System. Virus was recovered by transfecting a 1:1 ratio of replication cycle reaction product and pUC19^43^ into BHK-21 cells using Polyplus jetOPTIMUS DNA transfection reagent (101000051). Three days post-transfection, a blind passage of transfection supernatant on Vero E6-TMPRSS2-T2A-hACE2 cells was performed. After observing cytopathic effect, the virus was harvested, titered by plaque assay on Vero E6-TMPRSS2-T2A-hACE2 cells, and 400 bp libraries were prepared in-house using IDT Artic V4.1 NCOV-2019 Panel (10011442) and sequenced by Genewiz NGS Amplicon-EZ Illumina sequencing.

### Growth curve of Spike H519N in human lung epithelial cells

Human lung epithelial cells (Calu-3) were infected at 80% confluency with a MOI of 0.1 of SARS-CoV-2 Wuhan-Hu-1 (wild-type), SARS-CoV-2 Spike D614G mutant, and SARS-CoV-2 Spike H519N mutant diluted in Roswell Park Memorial Institute 1640 (RPMI-1640) medium supplemented with fetal bovine serum (2%) and HEPES (10 mM). Supernatant was harvested every 24 h post-infection and titered by plaque assays on Vero E6-TMPRSS2-T2A-hACE2 cells.

### Quantitation of infectious virus

Infectious virus was quantified by plaque assay by infecting 90% confluent monolayers of Vero E6-TMPRSS2-T2A-hACE2 with serial dilutions of virus made in RPMI-1640 medium supplemented with fetal bovine serum (2%) and HEPES (10 mM). After 1 h of adsorption at 37 °C, a 1:1 mixture of 2X media (2X EMEM, 4X L-glutamine, 0.735% sodium bicarbonate, 0.2 mg/mL gentamicin sulfate, 4% FBS, 20 mM HEPES) and 3% methylcellulose was added to the cells. Infection proceeded for three days at 37 °C before fixation in 10% buffered formalin and staining with a 0.1% crystal violet solution containing 20% ethanol to visualize plaques.

### Structural analyses

Molecular Mechanics/Generalized Born Surface Area (MM/GBSA) was used to predict interchain interaction energy with the Schrodinger-Maestro software v. 2020-2 (Schrödinger, LLC, New York, NY, 2020). Spike protein (PDB ID: 7KNB) was split into individual chains and modified to reflect D614G in the Wuhan-Hu-1 strain^44^. Predicted interaction energy was calculated between Spike chains first on one chain in an up-RBD conformation and one chain in a down-RBD conformation to approximate up conformation free energy and two down-RBD conformation chains for down conformation free energy on H519 (neutral), H519 (protonated), and N519 structures. Protonated and unprotonated states were simulated at a pH of 5 and 7, respectively, for H519. Analysis using IPC 2.0^45^ of the Spike sequence indicated that the region around H519 is mostly neutral with acidic propensity, suggesting the increased potential of an acidic environment and lower pH to promote a protonated H519. Surface mapping was performed using Schrodinger-Maestro software v. 2020-2.

### Biochemical characterization of Spike with human ACE2

A sandwich ELISA was performed using recombinant, purified RBD of His-tagged wild-type Wuhan-Hu-1 SARS-CoV-2, SARS-CoV-2 Spike N501Y, SARS-CoV-2 Spike H519N, or SARS-CoV-2 Spike A372T and hACE2. A plate was coated with hACE2-mFc (Cat:10108-H05H) at 2 μg/mL and incubated overnight at 4 °C. The following day, RBD of each aforementioned genotype was added at the following concentrations: 0.256, 1.28, 6.4, 32, 160, 800, and 4000 ng/mL. The antibody anti-His-HRP (Sino A5327) was used to detect the His-tagged RBDs. Absorbance was measured at 450 nm, and values were normalized to the blank.

### Statistical analyses

All statistical analyses for Figs. 2–3 were performed using Prism 9 (GraphPad). Growth curve data was analyzed using a two-way ANOVA with Dunnett’s correction for multiple comparisons. All other data were analyzed with a one-way ANOVA with Dunnett’s correction for multiple comparisons.

### Biosecurity

All research protocols were approved by the Virginia Tech Institutional Biosafety Committee prior to beginning experiments. Pseudotyped virus work was conducted at biosafety level 2, while live virus manipulation of SARS-CoV-2 was performed in a biosafety level 3 (BSL3) laboratory at Virginia Tech with appropriate fitting N95 respirators and other standard PPE. SARS-CoV-2 was appropriately inactivated at 65 °C for 10 min after the addition of a viral lysis buffer, as described previously, before removal from the BSL3 for library preparation^46^.

## Supplementary information

Supplementary Table

Supplementary Information

## Data Availability

Sequencing files of the viruses constructed in the current study are available from the corresponding author on reasonable request. All remaining data generated or analyzed during this study are included in this published article (and its supplementary information files).
