# Supplementary material for: Evolution at Spike protein position 519 in SARS-CoV-2 facilitated adaptation to humans
Source: Npj Viruses. 2024 Jul 9;2:29. doi: 10.1038/s44298-024-00036-2 (PMC11721114; doi:10.1038/s44298-024-00036-2)
Supplement: Supplementary file 2 — Supplementary Information [file 44298_2024_36_MOESM2_ESM.pdf]

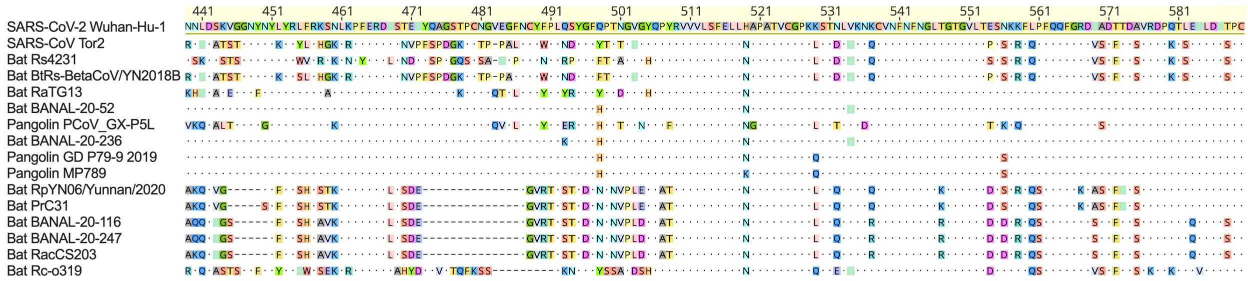

Supplementary Figure 1. Amino acid alignment of the full selective sweep region in Spike identified in January 2021.

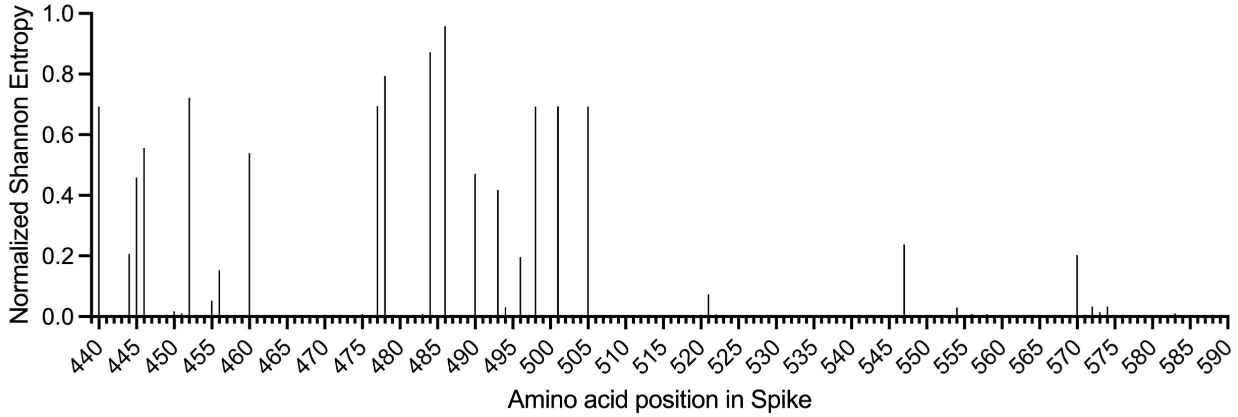

Supplementary Figure 2. Shannon Entropy in the selective sweep region in Spike of SARS-CoV-2. Amino acid diversity as represented by Shannon entropy normalized to the number of tips in the phylogenetic tree on Nextstrain for the sweep region where H519 was identified. Sequences on Nextstrain came from sequences uploaded to the GISAID database between December 2019 and October 2023.

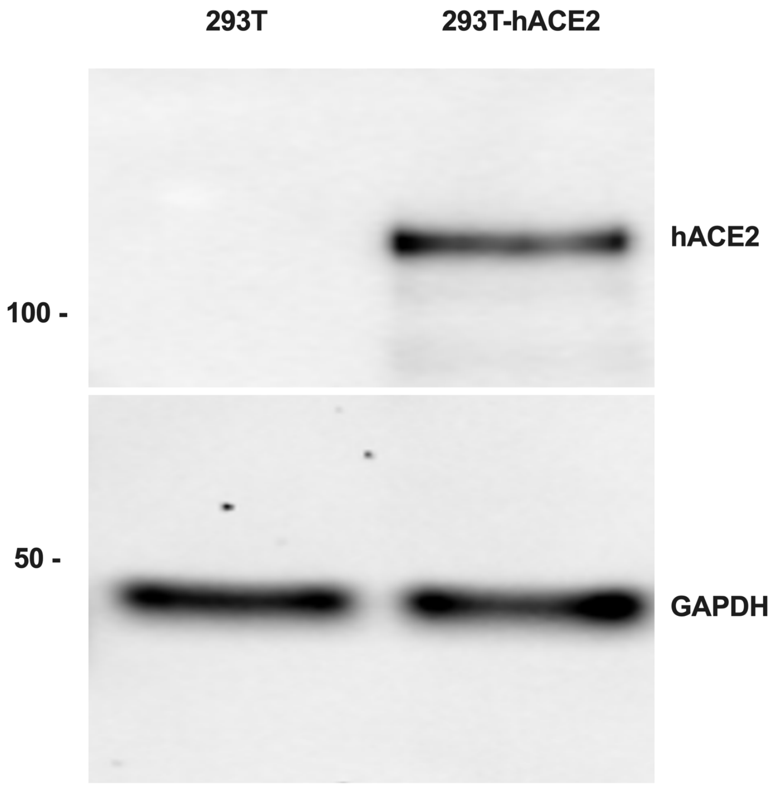

Supplementary Figure 3. **Expression of hACE2 by western blot on wild-type and human embryonic kidney cells engineered to express hACE2 (293T-hACE2).** Units are represented in kilodaltons (kDa).
